# Historical Blurry Video-Based Face Recognition

**DOI:** 10.3390/jimaging10090236

**Published:** 2024-09-20

**Authors:** Lujun Zhai, Suxia Cui, Yonghui Wang, Song Wang, Jun Zhou, Greg Wilsbacher

**Affiliations:** 1 Electrical and Computer Engineering Department, Prairie View A&M University, Prairie View, TX 77446, USA; lzhai@pvamu.edu (L.Z.); sucui@pvamu.edu (S.C.); 2 Computer Science Department, Prairie View A&M University, Prairie View, TX 77446, USA; 3 Computer Science and Engineering Department, University of South Carolina, Columbia, SC 29425, USA; songwang@cec.sc.edu (S.W.); zhouj@mailbox.sc.edu (J.Z.); 4 Moving Image Research Collections, University Libraries, University of South Carolina, Columbia, SC 29425, USA; gregw@mailbox.sc.edu

**Keywords:** face detection, face recognition, face tracking, historical blurry video

## Abstract

Face recognition is a widely used computer vision, which plays an increasingly important role in user authentication systems, security systems, and consumer electronics. The models for most current applications are based on high-definition digital cameras. In this paper, we focus on digital images derived from historical motion picture films. Historical motion picture films often have poorer resolution than modern digital imagery, making face detection a more challenging task. To approach this problem, we first propose a trunk–branch concatenated multi-task cascaded convolutional neural network (TB-MTCNN), which efficiently extracts facial features from blurry historical films by combining the trunk with branch networks and employing various sizes of kernels to enrich the multi-scale receptive field. Next, we build a deep neural network-integrated object-tracking algorithm to compensate for failed recognition over one or more video frames. The framework combines simple online and real-time tracking with deep data association (Deep SORT), and TB-MTCNN with the residual neural network (ResNet) model. Finally, a state-of-the-art image restoration method is employed to reduce the effect of noise and blurriness. The experimental results show that our proposed joint face recognition and tracking network can significantly reduce missed recognition in historical motion picture film frames.

## 1. Introduction

From the 1890s through the 1970s, still and moving image celluloid film was the dominant method for documenting the world and its inhabitants. The vaults of government, corporate, and university libraries continue to house unknown hours of moving image film that contain the movements of historically important persons. Face recognition and object-tracking technology can help preserve the appearance of such persons [1,2].

Face recognition methods vary from traditional handcrafted machine learning to increasingly popular deep neural networks trained with big datasets [3]. Face recognition can be categorized into two classes: still image-based face recognition (SIFR) and video-based face recognition (VFR) [4]. Images in SIFR are typically captured by professional photographers under good conditions. Thus, sufficient facial features can be extracted by a neural network model to detect faces. When the video frame tends to be very motion-blurry and faces in videos exhibit occlusions and richer pose variations, VFR can be used to overcome these challenges [5,6,7].

**Figure 1:**
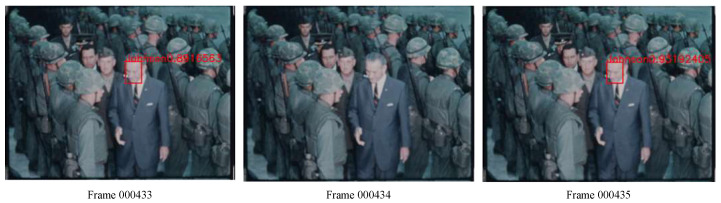
Face identification in historical motion picture video (President Johnson’s face can be identified, as indicated by the red boxes, with prediction probabilities in frames 000433 and 000435, while face identification failed in frame 000434).

Furthermore, historical videos have extrinsic features, noise, severe blurriness, etc., due to the low quality of video capture devices, which also exacerbates the lack of clarity in video frames [8,9]. These uncertain factors—noise, blurriness, lighting, occlusions, and pose variations—in historical video datasets can completely change the appearance of figures [10,11,12] and severely impact facial feature extraction, significantly increasing the difficulty in accurately recognizing a particular face and causing failure of face recognition in consecutive frames, as shown in Figure 1. Thus, recognizing the faces in these videos and tracking a particular figure can be very challenging. Therefore, it is critical to have an effective face detector for applications involving blurry videos.

State-of-the-art face detection methods, such as you only look once (YOLO) [13], joint face detection and alignment using the face as the center point (CenterFace) [14], light and fast face detector (LFFD) [15], single-shot scale-invariant face detector (S3FD) [16], Two-Stage cascaded convolutional neural network (Two-Stage CNN) [17], attribute-aware face proposal and face detection network (Faceness-Net) [18], contextual multi-scale region-based convolutional neural network (CMS-RCNN) [19], dual-shot face detector (DSFD) [20], six degrees of freedom and face image pose estimation without face detection or landmark localization (img2pose) [21], and multi-task cascaded convolutional neural network (MTCNN) [22], have achieved impressive performance on standard still images or high-quality videos captured by current popular digital cameras. However, these models suffer from severe performance degradation on blurry and low-resolution historical videos. Therefore, a major difficulty of face recognition in historical videos is largely unsolved.

The goal of this study is to build a neural network model with an object-tracking algorithm for historical video-based face recognition. Specifically, we propose a trunk–branch concatenated multi-task cascaded convolutional neural network (TB-MTCNN) to enhance face detection performance. The trunk network learns facial features from blurry historical film images, while the branch network learns facial features from the low-level feature map generated by the trunk network. Richer facial features can be extracted with multi-scale receptive fields by incorporating various sizes of convolution filters into the network. Low-level layers are shared by the trunk and branch networks to reduce computational cost. To form the comprehensive facial features, the feature maps output by the trunk and branch networks are fused through concatenation.

Another aspect of our method is that the image face detection algorithm may fail in some frames; we can recover such missed faces by tracking the same object in continuous frames. Specifically, the TB-MTCNN model is applied as a face detector to extract facial features and predict the face bounding box. Deep SORT [23], an object-tracking method integrating the Kalman filter, the intersection-over-union (IOU) distance algorithm, and the Hungarian data association algorithm, is applied to face tracking and face bounding box updating. Subsequently, the updated detected face bounding box is transferred to the residual neural network (ResNet) [24] classifier to recognize a particular face; finally, we obtain the historical figure’s face recognition. To better fit into face tracking, we optimize the object-based Deep SORT method utilizing the face-based Deep CNN model to efficiently extract facial features.

In addition, in order to reduce the effect of noise and blurriness and to fully extract facial features, we apply a state-of-the-art image restoration method, which brings old photos back to life [25] and is specifically designed for face restoration. The experimental results show that our proposed methods are promising in face detection in blurry videos and can effectively enhance face detection accuracy.

## 2. Related Works

### 2.1. Face Detection Using Deep Learning Algorithms

The concept of deep learning with neural networks was proposed many decades ago. Deep learning is a branch of machine learning methods based on artificial neural networks [26]. LeNet5 is the pioneering work in convolutional neural networks (CNNs), developed by researcher LeCun et al. in 1998 [27]. Due to the dramatic improvement in computing power and the explosion of big data, deep learning has made tremendous achievements over the past several years [28,29]. Problems related to sample scarcity can be solved by large datasets [30,31,32], and enhanced computing power has significantly accelerated the time-consuming training process. Deep learning-based approaches are increasingly applied in face detection fields and have significant advantages over traditional algorithms.

Region-based CNN methods have achieved state-of-the-art performance in generic object detection and classification [33], but satisfactory results have not been obtained in face detection. After training on a large-scale face detection dataset, the two-step approach Faster-RCNN [34], which employs a center loss function in the classification layer as an auxiliary signal, improved face detection performance to some extent. Faceness-Net [18] was proposed to address the issue of severe occlusions by leveraging facial attribute-based supervision. Img2pose [21] leverages the estimated 3D face pose to predict face bounding boxes. YOLO [13] and LFFD [15] mainly consider limited memory storage and low computing power and are designed for real-time edge device systems. Massively large receptive fields [35], as well as the EXTD [36] and S3FD [16] models, aim to find extremely small faces. Two-Stage CNN [17] is a relatively simple and coarse multi-scale proposal generation-based face detector. CMS-RCNN [19] was proposed to detect relatively small and heavily occluded faces by integrating body context. The cascaded CNN network [37] was proposed for fast face detection. The first level of the network uses the dense sliding window for window sampling on the entire picture for classification but cannot achieve satisfactory results in small face detection. The optimized multi-task cascaded architecture with three stages of deep convolutional networks, MTCNN [22], combines face detection and alignment and predicts face and landmark locations simultaneously. MTCNN enables any size of image input into the network and uses the convolution operation to substitute the sliding window, which improves the efficiency of network operations. Few works [38,39,40,41,42] have achieved favorable performance on still images or blur-free videos.

As described above, face detection models were proposed mainly to reduce network running time, address the small face detection problem, or enhance performance on relatively clear image datasets captured by modern digital cameras with high resolution and less noise. However, historical video datasets, captured by low-quality devices due to immature camera technology, exhibit features with severe image blur and low resolution. The face detector models used in most common VFR applications cannot achieve the desired results on historical blurry videos. To learn facial features more effectively from blurry video, we propose a novel CNN-based model, TB-MTCNN. It consists of one trunk network and several branch networks. The trunk network learns facial features from raw face images, and each branch network learns representations from low-level feature maps generated by the trunk network. By concatenating the output feature maps from the trunk and branch networks, the model obtains the fused comprehensive facial features. Compared to MTCNN, TB-MTCNN is more accurate when evaluated on historical blurry videos and two publicly available large-scale face datasets: CASIA-WebFace [43] and Wider Face [17].

### 2.2. Face Recognition Algorithms

Face detection algorithms are able to detect the presence of a human face in an image or video, while face recognition goes further to recognize whose face it is. After faces are detected in historical videos using the proposed TB-MTCNN model, we need to employ an image recognition algorithm to further classify and identify the detected faces.

Deep convolutional neural networks [27,44] have led to a series of breakthroughs in image classification. VGGNet [45] improved classification accuracy by deepening the weight layers of AlexNet [44] to 16–19, using unified small convolution filters, and employing the rectified linear unit (ReLU) activation function. GoogleNet [46], a 22-layer deep network with multiple sizes of convolution filters was proposed to allow for increasing the depth and width of the network while considering the limitations of computational resources. ResNet [24] has been widely used in image recognition, as it adapts residual representation learning and shortcut connections and enables deeper CNNs to be easier to train and avoid the degradation of training accuracy. In this paper, we adapt ResNet [24] with 18 layers as the face classifier to recognize a specific person from the human faces detected by TB-MTCNN.

### 2.3. Face-Tracking Algorithms

Although face detection has been well developed in real application scenes, camera motion and the quality of video somewhat degrade face detection results and cause missed detection problems in consecutive video frames. Thus, researchers have devised face-tracking techniques that aim to effectively and robustly utilize appearance or motion information from previous frames to estimate the object’s location in subsequent frames.

Many traditional methods like multiple hypothesis tracking (MHT) [47] and the joint probabilistic data association filter (JPDAF) [48] perform data association based on frame-by-frame information. Kim et al. proposed an appearance model for each target to prune the MHT graph [47]. In contrast, discriminative tracking methods like correlation filters (CFs) and kernelized correlation filters (KCFs) maintain a fixed target box throughout the tracking process [49]. However, changes in object sizes during the tracking process can lead to failures, as the correlation filters in these methods do not depend on detection information. Instead, they model the decision boundary and class differences. Predictive methods like the Kalman filter rely on detection information in the current frame and predict the most similar area in the next frame. SORT [50] and Deep SORT [23] employ the Kalman filter to anticipate facial positions in subsequent frames. Deep SORT enhances SORT by integrating appearance information, thereby improving tracking accuracy.

To efficiently account for missed facial recognition in consecutive video frames, we employ the Deep SORT algorithm as the face-tracking method in our proposed network.

### 2.4. Image Restoration

Old photo restoration is a classical mixed degradation problem. Most methods [51,52] focus on inpainting. Blurriness, noise, and blotches are identified based on low-level features and then inpainted using the textures nearby. Whyte proposed a non-uniform blind deblurring method based on a parametrized geometric model of the blurring process in terms of camera movement [53]. Noroozi employed a kernel-free end-to-end approach using a direct multi-scale CNN to deblur the images [54]. Image deblurring based on generative adversarial networks (GANs) [55,56,57] provided an effective tool to restore images and retrieve lost information. After deblurring, the recall rate of object detection increased on the synthetic datasets. However, these blind deblurring methods do not perform well on real-world images, as the blur is caused not only by the motion of different objects but also by camera jitter. To address this, a triplet-domain translation network was proposed [25], in which two variational autoencoders are trained to map old and clean photos into two separate latent spaces, effectively enhancing the visual quality of blurry photos to some extent. Ghosh explored the effects of nine kinds of degraded images and degradation removal on CNN-based image classification [58]. The degraded images decreased classification accuracy, while deblurring did not significantly improve classification accuracy.

Our work adapts one of the state-of-the-art image deblurring algorithms, which brings old photos back to life [25], to restore historical blurry videos and demonstrate the effectiveness of enhancing computer vision and face detection.

## 3. Face Recognition Structure

In this work, we make three main contributions to historical VFR. We propose a novel face detection neural network, TB-MTCNN, which efficiently extracts facial features from blurry historical videos by combining feature extraction from the trunk and branch networks. Further, to address the issue of missing detections, we integrate an object-tracking algorithm into the proposed face detection model. In addition, a state-of-the-art image restoration method is adopted to reduce the effects of noise and blurriness. The experimental results show that our proposed face recognition network significantly reduces missed detections in historical motion picture film frames, as detailed in Section 4.

**Figure 2:**
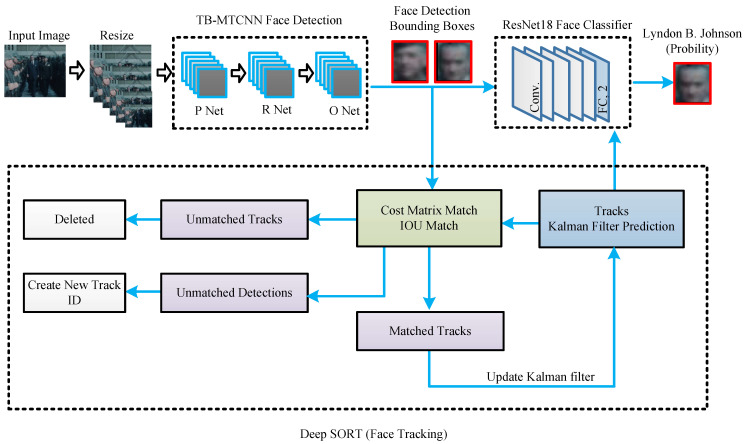
The architecture of the proposed model includes three components: a face detector (TB-MTCNN), a face tracker (Deep SORT), and a face classifier (ResNet18). First, TB-MTCNN processes the historical video frames to detect faces. Then, the Deep SORT algorithm utilizes face detection information from previous frames to track the face in the current frame if detection fails. Finally, the detection and tracking results are sent to the face classifier to determine whether the face belongs to President Johnson.

Our neural network model consists of three parts: detection, classification, and tracking. The proposed TB-MTCNN network is based on the MTCNN algorithm [22], which performs the face detection and face alignment tasks simultaneously. We adopt ResNet18, a variant of the ResNet architecture introduced by He et al. [24], as a face classifier to recognize a specific person. In our neural network design, we integrate the Deep SORT algorithm into our model to track the face. It uses face detection information from previous frames to predict the target location in the current frame. In this way, the accuracy of face recognition is greatly increased. The architecture of the proposed model, integrating detection, recognition, and tracking, is shown in Figure 2.

### 3.1. Face Detection

**Figure 3:**
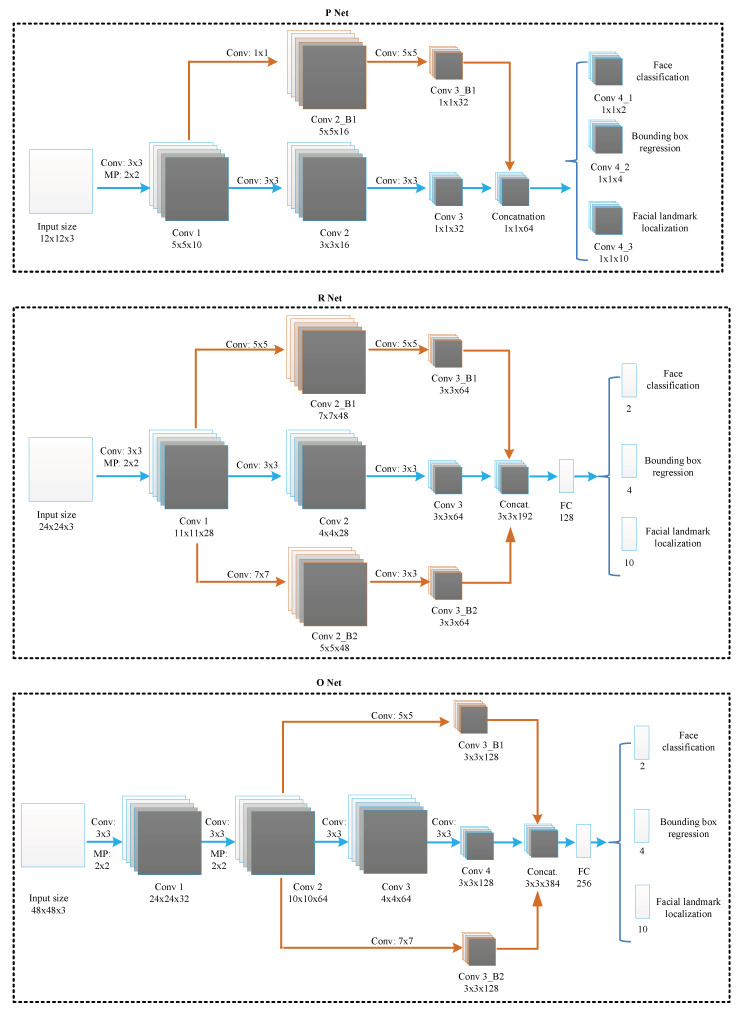
TB-MTCNN structure. The blue arrow represents the MTCNN architecture, while the yellow arrow highlights the integrated branch into the TB-MTCNN.

Face detection models used in common VFR applications cannot achieve the desired results in historical VFR, as video quality significantly affects face detection accuracy. To effectively extract rich facial features from historical videos, we propose a novel trunk–branch TB-MTCNN. The TB-MTCNN architecture is shown in Figure 3. The detailed parameters of our proposed TB-MTCNN model are shown in Table 1. The TB-MTCNN model consists of three sub-networks: P-Net, R-Net, and O-Net. These three stages of deep convolutional networks predict face and landmark locations using a coarse-to-fine method. The purpose of each sub-network is described as follows:

- P-Net (Proposal Network): P-Net is the first stage in the TB-MTCNN cascade and plays a crucial role in generating preliminary face candidates across the image. It scans the image using a sliding window approach, allowing for the detection of faces at various locations.
  - Multi-Scale Detection: To capture faces of different sizes, P-Net operates on multiple scaled versions of the input image, effectively enabling the detection of both small and large faces within a single frame.
  - Probabilistic Assessment: Along with proposing candidate bounding boxes, P-Net also assigns a probability score to each box, indicating the likelihood that it contains a face. This score is critical for subsequent filtering processes in later stages.
  - Input Image Size: The network processes input images of size 12×12 pixels, optimized for rapid detection and low computational overhead, making it suitable for real-time applications.
- R-Net (Refinement Network):
  - Refinement of Proposals: After the initial detection by P-Net, R-Net refines the candidate bounding boxes by more accurately discerning which contain faces, significantly reducing the number of false positives.
  - Enhanced Spatial Resolution: R-Net processes higher resolution images (24 × 24 pixels) compared to P-Net’s 12 × 12 pixels, enabling more effective discernment of facial features and improved localization of face boundaries.
  - Probability Filtering: R-Net computes a second-level confidence score to assess the likelihood of a face within each box, discarding boxes with low probability scores to reduce computational load and improve the precision of detections.
- O-Net (Output Network):
  - Final Bounding Box Adjustments: O-Net provides final adjustments to the bounding boxes, ensuring they are tightly fitted around the detected faces. This fine-tuning is crucial for applications requiring precise facial recognition or analysis.
  - Facial Landmark Detection: Beyond identifying faces, O-Net detects facial landmarks such as the eyes, nose, and mouth, supporting advanced facial analysis tasks like emotion recognition, facial alignment, and augmented reality applications.
  - High Receptive Field: With the largest input size (48 × 48 pixels), O-Net has a wider receptive field, allowing it to better integrate contextual information from a larger area of the image. This aids in accurate face and landmark localization even in complex scenarios.
  - Additional Outputs: The processing method of O-Net is similar to that of R-Net, but it also outputs face bounding box regressions and landmarks locations, in addition to performing face classification.

The trunk networks in each sub-network (colored in blue) take pyramid images as input and extract the coarse holistic facial features, while the branch networks (colored in brown) take the low-level feature maps generated by the trunk networks as input and further extract fine facial features. To extract facial features from multi-scale receptive fields, we integrate multiple sizes of convolution kernels into the branch networks, such as 1 × 1, 3 × 3, 5 × 5, and 7 × 7. By concatenating the output feature maps from the trunk and branch networks, the model obtains the fused, comprehensive facial features. Therefore, facial features can be extracted more completely.

We leverage three tasks to train the TB-MTCNN model: face classification, bounding box regression, and facial landmark localization. The learning objective is a two-class classification problem. We use the cross-entropy loss:

(1) $$L_{i}=-\left(y_{i}\log\left(p_{i}\right)+\left(1-y_{i}\right)\log\left(1-p_{i}\right)\right),$$

where $L_{i}$ is the loss for the *i*-th training example, $p_{i}$ is the probability predicted by the network that the i-th sample is a face, and $y_{i}$ denotes the ground-truth label. For the bounding box regression problem, we employ the Euclidean loss:

(2) $$L_{i}^{box}=\left|\right|{\hat{y}}_{i}^{box}-y_{i}^{box}{\left|\right|}_{2}^{2},$$

where ${\hat{y}}_{i}^{box}$ is the regression target predicted by our network and $y_{i}^{box}$ represents the ground-truth coordinates.

Facial landmark detection is also a regression problem, and we use the Euclidean loss:

(3) $$L_{i}^{landmark}=\left|\right|{\hat{y}}_{i}^{landmark}-y_{i}^{landmark}{\left|\right|}_{2}^{2},$$

where ${\hat{y}}_{i}^{landmark}$ is the facial landmark’s coordinates generated by the model and $y_{i}^{landmark}$ is the ground-truth coordinate value.

### 3.2. Face-Tracking Algorithm

To track faces in video frames, we adopt an object-tracking algorithm. This algorithm relies on face detection obtained from the current frame to predict face localization in the next frame. When the target fails to be detected by the TB-MTCNN model, the face-tracking algorithm can recover the missing face.

SORT (simple online and real-time tracking) [50] is a tracking method that employs Kalman filtering in image space and uses the Hungarian algorithm for optimal matching. Despite its efficiency, SORT struggles with occlusions, often assigning new track IDs to the same target when track continuity is temporarily lost. To enhance robustness against target losses, we incorporate the Deep SORT method. Deep SORT extends SORT by integrating a motion model with appearance information, allowing for more effective tracking of objects through occlusions and interactions. To enhance robustness against target losses, we incorporate the Deep SORT method. Deep SORT extends SORT by integrating a motion model with appearance information, allowing for more effective tracking of objects through occlusions and interactions. Deep SORT uses the cosine distance to weigh the appearance similarity between detections and tracks. To exclude the impossible condition, the Mahalanobis distance is used. Additionally, it adopts a matching cascade architecture to address the measurement-to-track associations, which increases robustness against target omissions and occlusions.

To accurately incorporate motion information, the squared Mahalanobis distance between predicted Kalman states and detections is used:

(4) $$d_{i,j}^{\left(1\right)}={\left(d_{j}-p_{i}\right)}^{T}S_{i}^{-1}\left(d_{j}-p_{i}\right),$$

where $d_{j}$ represents the position of the *j*-th bounding box detection and $p_{i}$ denotes the predicted position of the target from the *i*-th tracker. $S_{i}$ is the covariance matrix that quantifies the uncertainty between the detection position and the predicted tracking position. $d_{i,j}^{\left(1\right)}$ quantifies the squared Mahalanobis distance for the *i*-th tracker and the *j*-th detection, measuring the statistical distance between the predicted state and the actual detection under the assumption of a Gaussian distribution. The pair $\left(p_{i},S_{i}\right)$ represents the projection of the *i*-th track’s distribution into the measurement space. We set the threshold value of the Mahalanobis distance $t^{\left(1\right)}$ to 0.95 to exclude unlikely associations. $b_{i,j}^{\left(1\right)}$ indicates whether the association between the *i*-th tracker and the *j*-th detection is admissible:

(5) $$b_{i,j}^{\left(1\right)}=\left[d^{\left(1\right)}\left(i,j\right)\right]⩽t^{\left(1\right)}],$$

where $d_{i,j}^{\left(1\right)}$ denotes the squared Mahalanobis distance between the *i*-th tracker and the *j*-th detection, and $t^{\left(1\right)}$ represents the threshold value for the Mahalanobis distance to exclude unlikely associations between the tracker and detection.

To address scenarios where motion information may be less discriminative, such as after long-term occlusions, we adopt the cosine distance as a second metric. This is supported by the appearance descriptor $r_{j}$ for each detection $d_{j}$ , normalized to have a unit norm. We create a gallery $R_{k}={\left\{r_{k}^{i}\right\}}_{k=1}^{L_{k}}$ for each track *k*, consisting of the last 100 associated appearance descriptors. The second metric $d_{i,j}^{\left(2\right)}$ measures the smallest cosine distance between the *i*-th tracker and the *j*-th detection in the appearance space:

(6) $$d_{i,j}^{\left(2\right)}=\min\left\{1-r_{j}^{T}r_{k}^{i}|r_{k}^{i}\in R^{i}\right\}.$$

This approach leverages both motion and appearance information to improve tracking accuracy and identity recovery in complex scenarios.

To further refine face tracking, we adopt Deep SORT [23], which utilizes a Deep CNN appearance descriptor initially trained on the pedestrian dataset MARS [59]. Given our specific requirements for face tracking, we retrain the model using a face-based dataset rather than a person-based dataset, ensuring that the extracted features are optimally suited for our application. The training dataset was sourced from historical video footage, providing a robust basis for feature extraction.

### 3.3. Face Classifier

To complete the face classification task with two classes—President Lyndon B. Johnson and unknown—a face classifier ResNet18 [24] is integrated into our face recognition network. ResNet offers a significant advantage in that the residual connection or shortcut connection is built across the residual blocks to avoid overfitting, gradient explosion, and vanishing gradient problems in image classification [60]. ResNet18 performs the face classification task as follows: first, RestNet18 takes the faces predicted by TB-MTCNN as input. Then, the convolutional layers extract facial features. Lastly, the fully connected layer predicts the probability of the face being Johnson’s or unknown. In order to recognize the specific face, we fine-tuned the pre-trained ResNet model, which was trained on the labeled faces in the wild (LFW) [61] dataset and has extracted generic facial features. The reason we adapted fine-tuning is that our training dataset containing historical videos with President Johnson’s face is small. It is best to train only the classifier layer with this small dataset. The lower layers of the network contain generic facial features, while the top layers contain specific facial features. To fine-tune ResNet [24], we froze all the weights except for the last fully connected (FC) layer. This final layer was replaced with random weights, and only this layer was trained with our dataset to extract the specific facial features.

## 4. Experiments

### 4.1. Effectiveness of the Proposed TB-MTCNN Model

Training Dataset: The proposed TB-MTCNN model was trained on a synthesized blurry images dataset. Image degradation was implemented on the publicly available Wider Face [17] dataset. This dataset includes 393,703 labeled faces with a high degree of variability in scales, poses, and occlusions, and is organized based on 61 different kinds of events. The training dataset was composed of negative, positive, partial, and landmark faces. Negative and positive samples were used for face classification tasks; positive and partial faces were used for bounding box regression tasks; landmark faces were used for facial landmark localization tasks. The image degradation included Gaussian blur and noise. The range for the random Gaussian blur kernel width was set to [1, 25]. The noise consisted of Gaussian noise as well as salt and pepper noise. The variance of Gaussian noise was specified within the range from 0 to 29, which corresponds to 0 to 29 percent of the maximum possible pixel value range, ensuring a relative measure across different image intensities. Salt and pepper noise levels were set within the range of [0, 0.005], quantifying the proportion of pixels affected.

Testing Dataset: To evaluate the proposed TB-MTCNN face detection model, we first conducted experiments on two publicly available large-scale face datasets: Wider Face [17] and CASIA-WebFace [43]. Then, we tested the model on a historical video dataset. The video dataset was obtained from the United States Marine Corps Film Repository. The online collection can be found at https://digital.library.sc.edu/collections/united-states-marine-corps-films/ (accessed on 15 September 2020)

**Figure 4:**
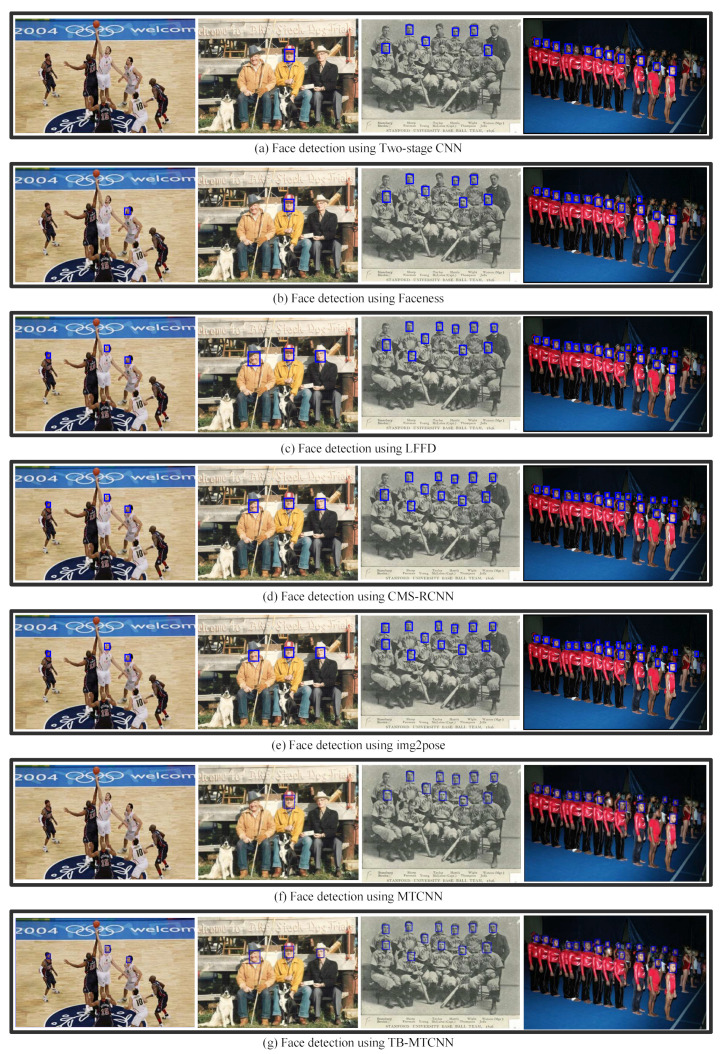
Performance comparison of face detection models on the Wider Face Medium dataset.

Performance Comparison on Wider Face [17]: This experiment evaluated the performance of the proposed TB-MTCNN model on the Wider Face Medium dataset. Figure 4 presents example images for the visual performance comparison between our approach and five state-of-the-art methods: Two-Stage CNN [17], Faceness [18], LFFD [15], CMS-RCNN [19], and img2pose [21]. Table 2 shows the quantitative results in terms of precision, recall, and F1 score. The proposed approach, TB-MTCNN, outperformed the original MTCNN model by as much as 7.3% in terms of the F1 score and achieved results comparable to the most advanced method, img2pose [21].

***Performance Comparison on CASIA-WebFace*** [43]: Next, we evaluated the performance of the face detection models on the CASIA-WebFace dataset, which contains 494,414 face images of 10,575 real identities collected from the web. Similar to the previous experiment, we applied these face detection models to the CASIA-WebFace testing dataset. Table 3 presents the performance comparison results. TB-MTCNN clearly outperformed the original MTCNN [22], Two-Stage CNN [17], and Faceness [18] in terms of the F1 score. The experimental results of TB-MTCNN, LFFD [15], CMS-RCNN [19], and img2pose [21] were very similar, but the performance of TB-MTCNN was slightly better than that of LFFD [15] and CMS-RCNN [19].

**Figure 5:**
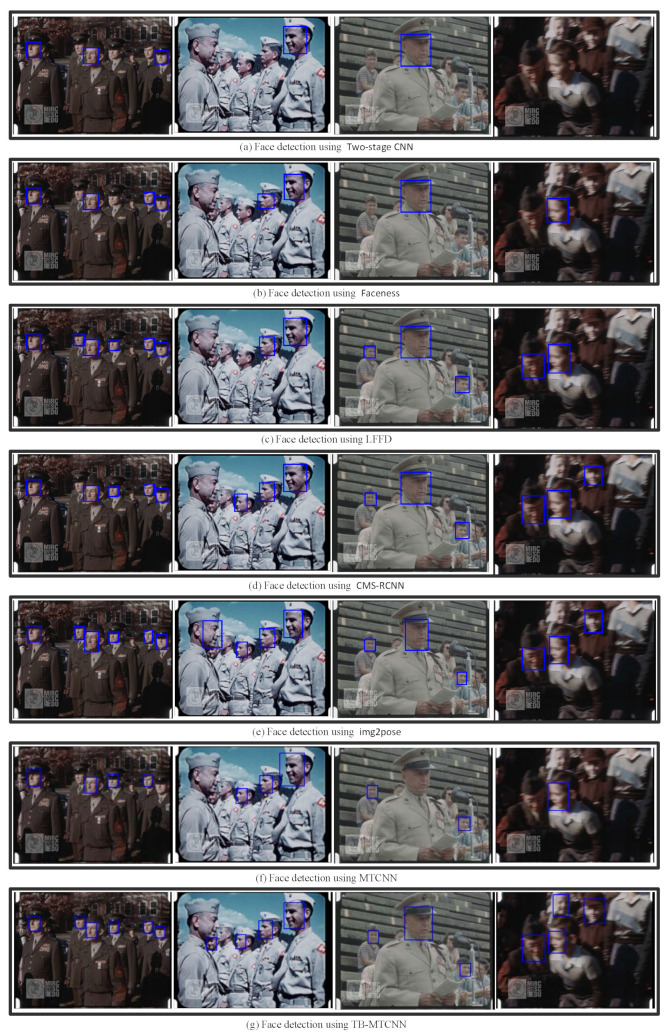
Sample performance comparison of face detection models on historical videos.

Performance Comparison on Historical Videos: Finally, we compared the performance of the face detection models on historical videos. The United States Marine Corps Film Repository provides a visual record of Marines during World War I and World War II. The faces in the film exhibit a high degree of variability in scales, poses, and occlusions. Figure 5 and Table 4 present a visual comparison of the performance on sample images and the quantitative results, respectively. TB-MTCNN outperformed MTCNN [22], Two-Stage CNN [17], Faceness [18], LFFD [15], and CMS-RCNN [19] and exhibited similar performance to the most advanced model, img2pose [21].

Experimental Results Analysis: The proposed TB-MTCNN and five state-of-the-art methods exhibited consistent performance results across three different datasets. Two-Stage CNN, a coarse multi-scale proposal generation-based face detector, lacks a refined process for predicting faces; therefore its performance significantly lagged behind that of TB-MTCNN and MTCNN [22]. Faceness [18] employs facial attribute supervision but lacks multi-scale facial feature extraction, leading to poorer performance in face detection. LFFD [15] and CMS-RCNN [19] achieved comparable results, but their performance was worse than that of TB-MTCNN. TB-MTCNN significantly outperformed the original MTCNN [22], as it combines the trunk and branch networks with multiple sizes of convolution kernels to enrich the receptive field and efficiently extract comprehensive facial features. Img2pose [21] slightly surpassed TB-MTCNN, as it adopts an indirect approach that leverages the estimated 3D face pose to achieve face detection. Compared to the relatively complex img2pose [21] algorithm, our proposed method is simple and can have a general application, making it easily integrable into all CNN-based face detection models. Specifically, branch networks with multiple sizes of convolution kernels, such as 1 × 1, 3 × 3, 5 × 5, and 7 × 7, can be introduced into CNN-based models. These, branch networks take coarse feature maps from the low-level layers of the trunk network and further extract fine facial features from multi-scale receptive fields using these multiple sizes of convolution kernels. Finally, comprehensive facial features are formed by fusing the outputs of the branch and trunk networks. In this paper, the trunk network implementation is based on MTCNN [22], and the simplicity and effectiveness of the proposed method have been demonstrated.

### 4.2. Fine-Tuning the Face Classifier

Collection of training and testing datasets: To train the face classifier ResNet18 [24] and recognize President Johnson’s face, we collected images of President Lyndon B. Johnson and those of other people around him from historical films. The training dataset included 2000 images of President Johnson’s face and 2000 images of unknown faces, while the testing data set included 500 images of President Johnson’s face and 500 images of unknown faces. All images were resized to 320×320 .

**Figure 6:**
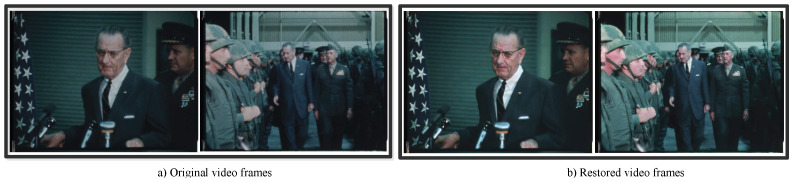
Effects of image restoration.

Considering that our dataset contained noise and blurriness, to reduce these effects and extract facial features more effectively, we applied a state-of-the-art restoration method, which brings old photos back to life [25], to restore the images in both the training and testing sets. After restoration, we observed that the image quality improved from a visual perspective, as shown in Figure 6.

We conducted ResNet18 fine-tuning on the original blurry image dataset and the restored image dataset. The best training accuracy of the neural network model using the original data was 0.9312, while the best training accuracy using the restored images was 0.9528. This demonstrates that face-based image restoration can significantly improve face identification accuracy.

### 4.3. Training the Deep CNN as a Face Appearance Descriptor

To train the Deep CNN model, we annotated the ID and location information in the historical video film to create the training dataset. The dataset was split into two sets: 75% for training and 25% for testing. We set the total training duration to 100 epochs. The initial learning rate was set to 0.1, and we applied a decay factor that reduced the learning rate by multiplying it by 0.1 every 5 epochs. This frequent decay strategy was designed to ensure a more dynamic adjustment of the learning rate, promoting better convergence throughout training. The training accuracy on our evaluation dataset reached 0.9588, and the loss was 0.04.

### 4.4. Face Recognition and Tracking Experiments

**Figure 7:**
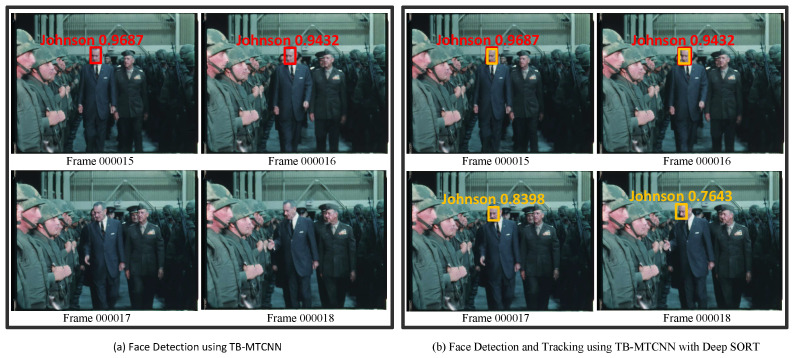
The Deep SORT method recovered the missed detections (the red boxes are faces detected using the TB-MTCNN detector, and the yellow boxes are faces recovered using Deep SORT).

Finally, we compared the historical VFR performance of TB-MTCNN with different approaches. The testing video film featured President Lyndon B. Johnson giving speeches and condolences to soldiers in the 1960s. We used the recall rate of President Johnson’s face to evaluate the performance of each method. The results in Figure 7 clearly show that by combining the Deep SORT algorithm and deblurring images, the recall rate was 0.879, representing the best performance among the detection and tracking methods.

The experimental results indicate that when face recognition fails, the tracking algorithm can still accurately capture President Johnson’s face position. For example, in Figure 7, we can see that using only the TB-MTCNN detector, President Johnson’s face in frames 000015 and 00016 was recognized, as indicated by the red bounding boxes, but it failed in the subsequent two frames. After integrating the Deep SORT approach, President Johnson’s face was tracked in frames 000017 and 000018, as indicated by the yellow bounding boxes. The object-tracking method successfully recovered the missing recognitions in these video frames. Table 5 shows the comparison results for various combinations of the proposed method.

## 5. Conclusions

Compared with SIFR and common VFR, historical VFR is challenging in the field of facial recognition because of its unique features like severe blurriness, noise, and low resolution. In this paper, we proposed an efficient face detection model by combining trunk and branch networks and integrating various sizes of convolution kernels into the network to enrich the receptive field. It was demonstrated that our proposed trunk–branch concatenated neural network significantly improves face detection accuracy across several different image datasets. Furthermore, we proposed a joint face recognition and tracking network for historical video-based face identification, which enhances robustness against target omissions and recovers missing recognition in consecutive frames. Moreover, the employed image restoration method effectively reduces the impact of noise and blurriness on face recognition. The presented network’s effectiveness makes it well suited for face recognition in historical VFR.

## Figures and Tables

**Table 1:** Parameters in the TB-MTCNN model.

P-net	in shape	in channels	out channels	kernel size	stride	padding	out shape
conv1	[batch,3,12,12]	3	10	3	1	0	[batch,10,10,10]
pool	[batch,10,10,10]	10	10	2	2	1	[batch,10,5,5]
conv2	[batch,10,5,5]	10	16	3	1	0	[batch,16,3,3]
conv2_B1	[batch,10,5,5]	10	16	1	1	0	[batch,16,5,5]
conv3	[batch,16,3,3]	16	32	3	1	0	[batch,32,1,1]
conv3_B1	[batch,16,5,5]	16	32	5	1	0	[batch,32,1,1]
conv4_1	[batch,32,1,1]	32	2	1	1	0	[batch,1,1,1]
conv4_2	[batch,32,1,1]	32	4	1	1	0	[batch,4,1,1]
conv4_3	[batch,32,1,1]	32	10	1	1	0	[batch,10,1,1]
R-net	in shape	in channels	out channels	kernel size	stride	padding	out shape
conv1	[batch,3,24,24]	3	28	3	1	0	[batch,28,22,22]
pool1	[batch,28,22,22]	28	28	3	2	1	[batch,28,11,11]
conv2	[batch,28,11,11]	28	48	3	1	0	[batch,48,9,9]
pool2	[batch,48,9,9]]	48	48	3	2	0	[batch,48,4,4]
conv2_B1	[batch,28,11,11]	28	48	5	1	0	[batch,48,7,7]
conv2_B2	[batch,28,11,11]	28	48	7	1	0	[batch,48,5,5]
conv3	[batch,48,4,4]	48	64	2	1	0	[batch,64,3,3]
conv3_B1	[batch,48,7,7]	48	64	5	1	0	[batch,64,3,3]
conv3_B2	[batch,48,5,5]	48	64	3	1	0	[batch,64,3,3]
line	in unit	out unit					
line1	192× 3× 3	128					
line2_1	128	1					
line2_2	128	4					
line2_3	128	10					
O-net	in shape	in channels	out channels	kernel size	stride	padding	out shape
conv1	[batch,3,48,48]	3	32	3	1	0	[batch,32,46,46]
pool1	[batch,32,46,46]]	32	32	2	2	1	[batch,32,24,24]
conv2	[batch,32,24,24]	32	64	3	1	0	[batch,64,22,22]
pool2	[batch,64,22,22]]	64	64	3	2	0	[batch,64,10,10]
conv3	[batch,64,10,10]	64	64	2	1	0	[batch,64,8,8]
pool3	[batch,64,8,8]]	64	64	2	2	0	[batch,64,4,4]
conv3_B1	[batch,128,10,10]	64	128	5	1	0	[batch,128,6,6]
pool3_B1	[batch,128,6,6]	128	128	2	2	0	[batch,128,3,3]
conv3_B2	[batch,64,10,10]	64	128	7	1	0	[batch,128,4,4]
pool3_B2	[batch,64,4,4]	128	128	2	1	0	[batch,128,3,3]
conv4	[batch,64,4,4]	64	128	2	1	0	[batch,128,3,3]
line	in unit	out unit					
line1	384 × 3 × 3	256					
line2_1	256	1					
line2_2	256	4					
line2_3	256	10					

**Table 2:** Performance comparison of face detection models on the Wider Face Medium dataset.

Method	Precision	Recall	F1 Score
Two-Stage CNN [17]	0.589	0.496	0.539
Faceness [18]	0.604	0.617	0.610
LFFD [15]	0.865	0.693	0.769
CMS-RCNN [19]	0.874	0.704	0.779
img2pose [21]	**0.891**	0.735	0.805
MTCNN [22]	0.820	0.636	0.716
TB-MTCNN (present)	0.883	0.713	0.789

**Table 3:** Performance comparison of face detection models on the CASIA-WebFace dataset.

Method	Precision	Recall	F1 Score
Two-Stage CNN [17]	0.750	0.631	0.686
Faceness [18]	0.807	0.716	0.759
LFFD [15]	0.938	0.940	0.939
CMS-RCNN [19]	0.943	0.937	0.940
img2pose [21]	0.962	0.950	0.956
MTCNN [22]	0.925	0.892	0.908
TB-MTCNN (present)	0.964	0.942	0.945

**Table 4:** Performance comparison of face detection models on historical videos.

Method	Precision	Recall	F1 Score
Two-Stage CNN [17]	0.517	0.192	0.280
Faceness [18]	0.548	0.206	0.299
LFFD [15]	0.829	0.315	0.457
CMS-RCNN [19]	0.831	0.319	0.461
img2pose [21]	0.842	0.336	0.480
MTCNN [22]	0.750	0.223	0.344
TB-MTCNN (present)	0.836	0.325	0.468

**Table 5:** Historical VFR performance comparison of different methods.

Method	Recall Rate	Precision
TB-MTCNN + ResNet	0.685	0.835
TB-MTCNN + ResNet + SORT	0.754	0.812
TB-MTCNN + ResNet + Deep SORT	0.863	0.834
TB-MTCNN + ResNet + Deep SORT+ Image Restoration	0.879	0.845

## Data Availability

Due to privacy concerns related to the historical films from the Moving Image Research Collections, the data involved in this research will not be released.
